# Supplementary material for: Application of Real-Time PCR for the Detection and Quantification of Oomycetes in Ornamental Nursery Stock
Source: J Fungi (Basel). 2021 Jan 27;7(2):87. doi: 10.3390/jof7020087 (PMC7911672; doi:10.3390/jof7020087)
Supplement: Supplementary file 1 [file jof-07-00087-s001.pdf]

Table S1. - Genbank accession numbers for the ITS region of oomycete isolates, MF115150 – MF115524.

DNA Sample Code	Accession Number	Isolate	Plant sample	Plant Species	Culture Collection	Source
ABD.1	MF115150	*Phytopythium litorale*	P1	*Viburnum* x *bodnantense* 'Dawn'	C001	Substrate
ABD.12	MF115151	*Pythium undulatum*	P3	*Acer palmatum* 'Orange dream'	C009	Baiting leaves
ABD.13	MF115152	*Phytophthora plurivora*	P4	*Acer palmatum* 'Orange dream'	C010	Baiting leaves
ABD.14	MF115153	*Pythium debaryanum*	P4	*Acer palmatum* 'Orange dream'	C011	Baiting leaves
ABD.16	**MF115154**	*Pythium undulatum*	P6	*Acer palmatum* 'Atropurpureum'	C012	Baiting leaves
ABD.17	MF115155	*Pythium torulosum*	P6	*Acer palmatum* 'Atropurpureum'	C013	Baiting leaves
ABD.18	MF115156	*Pythium intermedium*	P7	*Acer palmatum* 'Atropurpureum'	C014	Baiting leaves
ABD.19	MF115157	*Pythium intermedium*	P7	*Acer palmatum* 'Atropurpureum'	C015	Baiting leaves
ABD.2	MF115158	*Pythium dissotocum*	P1	*Viburnum* x *bodnantense* 'Dawn'	C002	Baiting leaves
ABD.20	MF115159	*Phytophthora ramorum*	P1	*Viburnum* x *bodnantense* 'Dawn'	C016	Substrate
ABD.21	MF115160	*Phytophthora ramorum*	P1	*Viburnum* x *bodnantense* 'Dawn'	C017	Substrate
ABD.22	MF115161	*Phytophthora ramorum*	P1	*Viburnum* x *bodnantense* 'Dawn'	C018	Substrate
ABD.23	MF115162	*Phytophthora cryptogea*	P1	*Viburnum* x *bodnantense* 'Dawn'	C019	Baiting leaves
ABD.24	MF115163	*Phytophthora cryptogea*	P1	*Viburnum* x *bodnantense* 'Dawn'	C020	Baiting leaves
ABD.25	MF115164	*Phytophthora ramorum*	P1	*Viburnum* x *bodnantense* 'Dawn'	C021	Roots
ABD.26	MF115165	*Phytophthora ramorum*	P1	*Viburnum* x *bodnantense* 'Dawn'	C022	Baiting leaves
ABD.28	MF115166	*Phytophthora plurivora*	P4	*Acer palmatum* 'Orange dream'	C023	Substrate
ABD.29	MF115167	*Phytophthora plurivora*	P4	*Acer palmatum* 'Orange dream'	C024	Baiting leaves
ABD.3	MF115168	*Pythium dissotocum*	P1	*Viburnum* x bodnantense 'Dawn'	C003	Baiting leaves
ABD.30	MF115169	*Phytophthora ramorum*	P1	*Viburnum* x bodnantense 'Dawn'	C025	Substrate
ABD.4	MF115170	*Pythium dissotocum*	P1	*Viburnum* x bodnantense 'Dawn'	N	Baiting leaves
ABD.5	MF115171	*Pythium dissotocum*	P1	*Viburnum* x bodnantense 'Dawn'	N	Baiting leaves
ABD.7	MF115172	*Pythium irregulare*	P2	*Acer palmatum* 'Orange dream'	C005	Baiting leaves
ABD.8	MF115173	*Pythium dissotocum*	P2	*Acer palmatum* 'Orange dream'	C006	Baiting leaves
ABD.31	MF115174	*Phytophthora ramorum*	P1	*Viburnum* x bodnantense 'Dawn'	C029	Substrate
ABD.32	MF115175	*Phytophthora ramorum*	P1	*Viburnum* x bodnantense 'Dawn'	C026	Baiting leaves
ABD.33	MF115176	*Phytophthora ramorum*	P1	*Viburnum* x bodnantense 'Dawn'	C027	Baiting leaves
ABD.34	MF115177	*Phytophthora ramorum*	P1	*Viburnum* x bodnantense 'Dawn'	C028	Baiting leaves
ABD.35	MF115178	*Pythium dissotocum*	P8	*Hebe* x *franciscana* 'Variegata'	C030	Roots
ABD.36	MF115179	*Pythium dissotocum*	P8	*Hebe* x *franciscana* 'Variegata'	N	Roots
ABD.37	MF115180	*Pythium dissotocum*	P8	*Hebe* x *franciscana* 'Variegata'	C031	Baiting leaves
ABD.38	MF115181	*Phytopythium chamaehyphon*	P8	*Hebe* x *franciscana* 'Variegata'	C032	Substrate
ABD.39	MF115182	*Pythium dissotocum*	P8	*Hebe* x *franciscana* 'Variegata'	C033	Baiting leaves
ABD.40	MF115183	*Phytopythium litorale*	P9	*Hebe* x *franciscana* 'Variegata'	C034	Baiting leaves
ABD.41	MF115184	*Pythium dissotocum*	P9	*Hebe* x *franciscana* 'Variegata'	C035	Baiting leaves

ABD.42	MF115185	*Phytopythium chamaehyphon*	P9	*Hebe* x *franciscana* 'Variegata'	C036	Substrate
ABD.43	MF115186	*Phytopythium litorale*	P9	*Hebe* x *franciscana* 'Variegata'	C037	Substrate
ABD.44	MF115187	*Phytopythium litorale*	P9	*Hebe* x *franciscana* 'Variegata'	C038	Substrate
ABD.45	MF115188	*Pythium dissotocum*	P10	*Hebe* x *franciscana* 'Variegata'	C039	Roots
ABD.46	MF115189	*Phytopythium vexans*	P10	*Hebe* x *franciscana* 'Variegata'	C040	Baiting leaves
ABD.47	MF115190	*Pythium dissotocum*	P10	*Hebe* x *franciscana* 'Variegata'	C041	Baiting leaves
ABD.48	MF115191	*Phytopythium chamaehyphon*	P10	*Hebe* x *franciscana* 'Variegata'	C042	Substrate
ABD.49	MF115192	*Phytopythium vexans*	P10	*Hebe* x *franciscana* 'Variegata'	C043	Substrate
ABD.50	MF115193	*Pythium dissotocum*	P10	*Hebe* x *franciscana* 'Variegata'	C044	Baiting leaves
ABD.51	MF115194	*Pythium dissotocum*	P11	*Hebe* x *franciscana* 'Variegata'	C045	Substrate
ABD.52	MF115195	*Phytopythium litorale*	P11	*Hebe* x *franciscana* 'Variegata'	C046	Substrate
ABD.53	MF115196	*Pythium dissotocum*	P11	*Hebe* x *franciscana* 'Variegata'	C047	Baiting leaves
ABD.54	MF115197	*Pythium dissotocum*	P11	*Hebe* x *franciscana* 'Variegata'	N	Baiting leaves
ABD.55	MF115198	*Pythium dissotocum*	P12	*Hebe* x *franciscana* 'Variegata'	C048	Substrate
ABD.56	MF115199	*Phytopythium chamaehyphon*	P12	*Hebe* x *franciscana* 'Variegata'	C049	Substrate
ABD.57	MF115200	*Pythium dissotocum*	P12	*Hebe* x *franciscana* 'Variegata'	C050	Baiting leaves
ABD.58	MF115201	*Pythium dissotocum*	P13	*Hebe* x *franciscana* 'Variegata'	C051	Baiting leaves
ABD.59	MF115202	*Phytopythium chamaehyphon*	P13	*Hebe* x *franciscana* 'Variegata'	C052	Substrate
ABD.60	MF115203	*Pythium dissotocum*	P13	*Hebe* x *franciscana* 'Variegata'	C053	Baiting leaves
ABD.100	MF115204	*Pythium dissotocum*	P27	*Viburnum plicatum* 'Lanarth'	C067	Roots
ABD.61	MF115205	*Phytophthora cinnamomi*	P14	*Rhododendron* 'Germania'	C054	Roots
ABD.62	MF115206	*Phytophthora cinnamomi*	P14	*Rhododendron* 'Germania'	C055	Roots
ABD.64	MF115207	*Phytophthora cinnamomi*	P14	*Rhododendron* 'Germania'	C056	Baiting leaves
ABD.65	MF115208	*Phytophthora cinnamomi*	P14	*Rhododendron* 'Germania'	C057	Baiting leaves
ABD.66	MF115209	*Pythium undulatum*	P14	*Rhododendron* 'Germania'	C058	Baiting leaves
ABD.67	MF115210	*Pythium undulatum*	P14	*Rhododendron* 'Germania'	N	Substrate
ABD.68	MF115211	*Phytophthora cinnamomi*	P15	*Rhododendron* 'Germania'	C059	Roots
ABD.69	MF115212	*Phytophthora cinnamomi*	P15	*Rhododendron* 'Germania'	C060	Roots
ABD.70	MF115213	*Phytophthora cinnamomi*	P15	*Rhododendron* 'Germania'	C061	Baiting leaves
ABD.71	MF115214	*Pythium undulatum*	P15	*Rhododendron* 'Germania'	C062	Baiting leaves
ABD.72	MF115215	*Pythium undulatum*	P15	*Rhododendron* 'Germania'	N	Baiting leaves
ABD.74	MF115216	*Phytophthora cinnamomi*	P15	*Rhododendron* 'Germania'	C063	Substrate
ABD.75	MF115217	*Pythium macrosporum*	P16	*Rhododendron* 'Marcel Menard'	C064	Baiting leaves
ABD.77	MF115218	*Pythium undulatum*	P17	*Rhododendron* 'Marcel Menard'	C066	Baiting leaves
ABD.78	MF115219	*Pythium undulatum*	P17	*Rhododendron* 'Marcel Menard'	N	Substrate
ABD.79	MF115220	*Pythium macrosporum*	P17	*Rhododendron* 'Marcel Menard'	N	Substrate
ABD.80	MF115221	*Pythium undulatum*	P18	*Rhododendron* 'Percy Wiseman'	N	Baiting leaves
ABD.81	MF115222	*Pythium undulatum*	P18	*Rhododendron* 'Percy Wiseman'	C068	Substrate
ABD.82	MF115223	*Pythium undulatum*	P19	*Rhododendron* 'Percy Wiseman'	C069	Substrate

ABD.83	MF115224	*Pythium undulatum*	P19	*Rhododendron* 'Percy Wiseman'	N	Substrate
ABD.84	MF115225	*Pythium dissotocum*	P21	*Acer palmatum* 'Dissectum Filigree'	C070	Baiting leaves
ABD.85	MF115226	*Pythium dissotocum*	P21	*Acer palmatum* 'Dissectum Filigree'	N	Baiting leaves
ABD.86	MF115227	*Pythium dissotocum*	P21	*Acer palmatum* 'Dissectum Filigree'	N	Baiting leaves
ABD.87	MF115228	*Pythium dissotocum*	P22	*Acer palmatum* 'Dissectum Filigree'	C071	Baiting leaves
ABD.88	MF115229	*Pythium dissotocum*	P22	*Acer palmatum* 'Dissectum Filigree'	N	Baiting leaves
ABD.89	MF115230	*Pythium dissotocum*	P22	*Acer palmatum* 'Dissectum Filigree'	N	Roots
ABD.90	MF115231	*Phytophthora cambivora*	P22	*Acer palmatum* 'Dissectum Filigree'	C072	Roots
ABD.91	MF115232	*Phytophthora cambivora*	P22	*Acer palmatum* 'Dissectum Filigree'	C073	Roots
ABD.92	MF115233	*Phytophthora cambivora*	P22	*Acer palmatum* 'Dissectum Filigree'	C074	Roots
ABD.93	MF115234	*Phytophthora cambivora*	P23	*Acer palmatum* 'Dissectum Filigree'	C075	Roots
ABD.94	MF115235	*Phytophthora cambivora*	P23	*Acer palmatum* 'Dissectum Filigree'	C076	Roots
ABD.95	MF115236	*Pythium perplexum*	P23	*Acer palmatum* 'Dissectum Filigree'	N	Roots
ABD.96	MF115237	*Pythium dissotocum*	P23	*Acer palmatum* 'Dissectum Filigree'	C077	Baiting leaves
ABD.97	MF115238	*Phytophthora cambivora*	P24	*Acer palmatum* 'Dissectum Filigree'	C078	Roots
ABD.99	MF115239	*Pythium dissotocum*	P24	*Acer palmatum* 'Dissectum Filigree'	N	Roots
ABD.101	MF115240	*Pythium undulatum*	P14	*Rhododendron* 'Germania'	N	Baiting leaves
ABD.102	MF115241	*Phytophthora cinnamomi*	P15	*Rhododendron* 'Germania'	C080	Baiting leaves
ABD.103	MF115242	*Pythium undulatum*	P15	*Rhododendron* 'Germania'	N	Baiting leaves
ABD.105	MF115243	*Pythium undulatum*	P17	*Rhododendron* 'Marcel Menard'	N	Baiting leaves
ABD.106	MF115244	*Pythium undulatum*	P18	*Rhododendron* 'Percy Wiseman'	N	Baiting leaves
ABD.107	MF115245	*Pythium undulatum*	P19	*Rhododendron* 'Percy Wiseman'	N	Baiting leaves
ABD.108	MF115246	*Pythium dissotocum*	P20	*Acer palmatum* 'Dissectum Filigree'	C081	Baiting leaves
ABD.110	MF115247	*Phytophthora cambivora*	P20	*Acer palmatum* 'Dissectum Filigree'	C082	Substrate
ABD.111	MF115248	*Phytophthora cambivora*	P20	*Acer palmatum* 'Dissectum Filigree'	C083	Substrate
ABD.112	MF115249	*Phytophthora cambivora*	P21	*Acer palmatum* 'Dissectum Filigree'	C084	Roots
ABD.113	MF115250	*Phytophthora cambivora*	P21	*Acer palmatum* 'Dissectum Filigree'	C085	Roots
ABD.114	MF115251	*Pythium dissotocum*	P21	*Acer palmatum* 'Dissectum Filigree'	N	Baiting leaves
ABD.115	MF115252	*Phytophthora cambivora*	P21	*Acer palmatum* 'Dissectum Filigree'	C086	Baiting leaves
ABD.116	MF115253	*Pythium dissotocum*	P21	*Acer palmatum* 'Dissectum Filigree'	N	Substrate
ABD.117	MF115254	*Phytophthora cambivora*	P21	*Acer palmatum* 'Dissectum Filigree'	C087	Substrate
ABD.118	MF115255	*Pythium dissimile*	P22	*Acer palmatum* 'Dissectum Filigree'	C088	Roots
ABD.119	MF115256	*Pythium dissotocum*	P22	*Acer palmatum* 'Dissectum Filigree'	N	Baiting leaves
ABD.120	MF115257	*Pythium lutarium*	P22	*Acer palmatum* 'Dissectum Filigree'	N	Baiting leaves
ABD.121	MF115258	*Pythium dissotocum*	P22	*Acer palmatum* 'Dissectum Filigree'	N	Substrate
ABD.122	MF115259	*Phytophthora cambivora*	P22	*Acer palmatum* 'Dissectum Filigree'	C089	Substrate
ABD.123	MF115260	*Pythium dissotocum*	P23	*Acer palmatum* 'Dissectum Filigree'	N	Baiting leaves
ABD.124	MF115261	*Pythium intermedium*	P23	*Acer palmatum* 'Dissectum Filigree'	C090	Substrate
ABD.125	MF115262	*Phytophthora cambivora*	P23	*Acer palmatum* 'Dissectum Filigree'	C091	Substrate

ABD.126	MF115263	*Pythium dissotocum*	P24	*Acer palmatum* 'Dissectum Filigree'	N	Baiting leaves
ABD.128	MF115264	*Phytophthora cambivora*	P24	*Acer palmatum* 'Dissectum Filigree'	C092	Substrate
ABD.129	MF115265	*Pythium dissotocum*	P26	*Viburnum plicatum* 'Lanarth'	C093	Baiting leaves
ABD.130	MF115266	*Pythium intermedium*	P27	*Viburnum plicatum* 'Lanarth'	C094	Substrate
ABD.131	MF115267	*Pythium dissotocum*	P28	*Viburnum plicatum* 'Lanarth'	N	Baiting leaves
ABD.132	MF115268	*Pythium sterilum*	P29	*Viburnum plicatum* 'Lanarth'	C095	Baiting leaves
ABD.133	MF115269	*Pythium kashmirense*	P29	*Viburnum plicatum* 'Lanarth'	C096	Baiting leaves
ABD.134	MF115270	*Pythium kashmirense*	P29	*Viburnum plicatum* 'Lanarth'	C097	Baiting leaves
ABD.135	MF115271	*Phytopythium helicoides*	P29	*Viburnum plicatum* 'Lanarth'	C098	Substrate
ABD.136	MF115272	*Phytophthora cryptogea*	P30	*Viburnum burkwoodii* 'Park Farm hybrid'	C099	Roots
ABD.137	MF115273	*Phytophthora cryptogea*	P31	*Viburnum burkwoodii* 'Park Farm hybrid'	C100	Roots
ABD.138	MF115274	*Pythium dissotocum*	P31	*Viburnum burkwoodii* 'Park Farm hybrid'	C101	Baiting leaves
ABD.139	MF115275	*Phytophthora cryptogea*	P32	*Viburnum burkwoodii* 'Park Farm hybrid'	C102	Roots
ABD.140	MF115276	*Phytophthora cryptogea*	P32	*Viburnum burkwoodii* 'Park Farm hybrid'	C103	Baiting leaves
ABD.141	MF115277	*Pythium dissotocum*	P32	*Viburnum burkwoodii* 'Park Farm hybrid'	N	Baiting leaves
ABD.142	MF115278	*Phytophthora cryptogea*	P33	*Viburnum burkwoodii* 'Park Farm hybrid'	C104	Roots
ABD.143	MF115279	*Pythium anandrum*	P33	*Viburnum burkwoodii* 'Park Farm hybrid'	C105	Substrate
ABD.144	MF115280	*Phytophthora cryptogea*	P34	*Viburnum burkwoodii* 'Park Farm hybrid'	C106	Roots
ABD.145	MF115281	*Pythium dissotocum*	P34	*Viburnum burkwoodii* 'Park Farm hybrid'	C107	Baiting leaves
ABD.146	MF115282	*Phytophthora cryptogea*	P34	*Viburnum burkwoodii* 'Park Farm hybrid'	C108	Baiting leaves
ABD.147	MF115283	*Phytopythium litorale*	P34	*Viburnum burkwoodii* 'Park Farm hybrid'	C109	Substrate
ABD.148	MF115284	*Phytophthora cryptogea*	P34	*Viburnum burkwoodii* 'Park Farm hybrid'	C110	Substrate
ABD.149	MF115285	*Pythium dissotocum*	P35	*Viburnum tinus* 'Eve Price'	N	Baiting leaves
ABD.150	MF115286	*Phytopythium vexans*	P35	*Viburnum tinus* 'Eve Price'	C111	Substrate
ABD.151	MF115287	*Pythium dissotocum*	P36	*Viburnum tinus* 'Eve Price'	N	Baiting leaves
ABD.152	MF115288	*Pythium dissotocum*	P36	*Viburnum tinus* 'Eve Price'	N	Baiting leaves
ABD.154	MF115289	*Pythium dissotocum*	P38	*Viburnum tinus* 'Eve Price'	N	Baiting leaves
ABD.155	MF115290	*Phytophthora citrophthora*	P38	*Viburnum tinus* 'Eve Price'	C113	Substrate
ABD.156	MF115291	*Phytophthora citrophthora*	P38	*Viburnum tinus* 'Eve Price'	C114	Substrate
ABD.157	MF115292	*Pythium dissotocum*	P38	*Viburnum tinus* 'Eve Price'	N	Baiting leaves
ABD.158	MF115293	*Pythium dissotocum*	P39	*Viburnum tinus* 'Eve Price'	N	Baiting leaves
ABD.159	MF115294	*Pythium dissotocum*	P39	*Viburnum tinus* 'Eve Price'	N	Baiting leaves
ABD.160	MF115295	*Phytopythium chamaehyphon*	P39	*Viburnum tinus* 'Eve Price'	C115	Substrate
ABD.161	MF115296	*Phytopythium chamaehyphon*	P39	*Viburnum tinus* 'Eve Price'	C116	Substrate
ABD.162	MF115297	*Phytophthora cambivora*	P20	*Acer palmatum* 'Dissectum Filigree'	C117	Baiting leaves
ABD.163	MF115298	*Phytophthora cambivora*	P21	*Acer palmatum* 'Dissectum Filigree'	C118	Baiting leaves
ABD.164	MF115299	*Phytophthora plurivora*	P21	*Acer palmatum* 'Dissectum Filigree'	C119	Baiting leaves
ABD.165	MF115300	*Phytophthora cambivora*	P22	*Acer palmatum* 'Dissectum Filigree'	C120	Baiting leaves
ABD.166	MF115301	*Phytophthora cambivora*	P23	*Acer palmatum* 'Dissectum Filigree'	C121	Substrate

ABD.167	MF115302	*Pythium deliense*	P28	*Viburnum plicatum* 'Lanarth'	C122	Roots
ABD.168	MF115303	*Pythium kashmirense*	P29	*Viburnum plicatum* 'Lanarth'	C123	Baiting leaves
ABD.169	MF115304	*Phytopythium litorale*	P30	*Viburnum burkwoodii* 'Park Farm hybrid'	C124	Baiting leaves
ABD.170	MF115305	*Pythium dissotocum*	P30	*Viburnum burkwoodii* 'Park Farm hybrid'	C125	Baiting leaves
ABD.171	MF115306	*Phytophthora taxon PgChlamydo*	P30	*Viburnum burkwoodii* 'Park Farm hybrid'	C126	Baiting leaves
ABD.172	MF115307	*Phytophthora cryptogea*	P30	*Viburnum burkwoodii* 'Park Farm hybrid'	C127	Baiting leaves
ABD.173	MF115308	*Phytopythium litorale*	P30	*Viburnum burkwoodii* 'Park Farm hybrid'	C128	Substrate
ABD.174	MF115309	*Phytophthora cryptogea*	P30	*Viburnum burkwoodii* 'Park Farm hybrid'	C129	Substrate
ABD.175	MF115310	*Phytophthora cryptogea*	P31	*Viburnum burkwoodii* 'Park Farm hybrid'	C130	Baiting leaves
ABD.176	MF115311	*Phytopythium litorale*	P31	*Viburnum burkwoodii* 'Park Farm hybrid'	C131	Baiting leaves
ABD.177	MF115312	*Phytophthora cryptogea*	P31	*Viburnum burkwoodii* 'Park Farm hybrid'	C132	Substrate
ABD.178	MF115313	*Phytophthora cryptogea*	P32	*Viburnum burkwoodii* 'Park Farm hybrid'	C134	Substrate
ABD.179	MF115314	*Phytophthora cryptogea*	P32	*Viburnum burkwoodii* 'Park Farm hybrid'	C133	Baiting leaves
ABD.180	MF115315	*Phytophthora cryptogea*	P33	*Viburnum burkwoodii* 'Park Farm hybrid'	C136	Baiting leaves
ABD.181	MF115316	*Phytophthora cryptogea*	P33	*Viburnum burkwoodii* 'Park Farm hybrid'	C135	Substrate
ABD.182	MF115317	*Phytophthora taxon PgChlamydo*	P34	*Viburnum burkwoodii* 'Park Farm hybrid'	C137	Substrate
ABD.183	MF115318	*Phytophthora cactorum*	P34	*Viburnum burkwoodii* 'Park Farm hybrid'	C138	Substrate
ABD.184	MF115319	*Phytophthora citrophthora*	P35	*Viburnum tinus* 'Eve Price'	C139	Baiting leaves
ABD.188	MF115320	*Phytophthora cactorum*	P37	*Viburnum tinus* 'Eve Price'	C140	Substrate
ABD.189	MF115321	*Phytopythium chamaehyphon*	P37	*Viburnum tinus* 'Eve Price'	C141	Substrate
ABD.190	MF115322	*Phytophthora cactorum*	P37	*Viburnum tinus* 'Eve Price'	C142	Substrate
ABD.191	MF115323	*Phytophthora citrophthora*	P38	*Viburnum tinus* 'Eve Price'	C143	Baiting leaves
ABD.192	MF115324	*Phytophthora cryptogea*	P38	*Viburnum tinus* 'Eve Price'	C144	Baiting leaves
ABD.194	MF115325	*Phytophthora cactorum*	P39	*Viburnum tinus* 'Eve Price'	C145	Baiting leaves
ABD.197	MF115326	*Phytophthora taxon PgChlamydo*	P33	*Viburnum burkwoodii* 'Park Farm hybrid'	C146	Baiting leaves
ABD.198	MF115327	*Phytophthora taxon PgChlamydo*	P33	*Viburnum burkwoodii* 'Park Farm hybrid'	C147	Baiting leaves
ABD.199	MF115328	*Phytophthora cryptogea*	P34	*Viburnum burkwoodii* 'Park Farm hybrid'	C148	Baiting leaves
ABD.200	MF115329	*Phytophthora cactorum*	P35	*Viburnum tinus* 'Eve Price'	C149	Baiting leaves
ABD.201	MF115330	*Phytophthora cactorum*	P35	*Viburnum tinus* 'Eve Price'	N	Baiting leaves
ABD.202	MF115331	*Phytophthora cactorum*	P35	*Viburnum tinus* 'Eve Price'	C150	Baiting leaves
ABD.203	MF115332	*Phytophthora cactorum*	P37	*Viburnum tinus* 'Eve Price'	C151	Baiting leaves
ABD.204	MF115333	*Phytophthora cactorum*	P37	*Viburnum tinus* 'Eve Price'	C152	Baiting leaves
ABD.205	MF115334	*Phytophthora cactorum*	P37	*Viburnum tinus* 'Eve Price'	N	Baiting leaves
ABD.206	MF115335	*Phytophthora cactorum*	P39	*Viburnum tinus* 'Eve Price'	N	Baiting leaves
ABD.207	MF115336	*Phytophthora cactorum*	P39	*Viburnum tinus* 'Eve Price'	C153	Baiting leaves
ABD.208	MF115337	*Phytophthora cactorum*	P39	*Viburnum tinus* 'Eve Price'	C154	Baiting leaves
ABD.212	MF115338	*Pythium heterothallicum*	P43	*Camellia alba* 'Plena'	C155	Roots
ABD.214	MF115339	*Pythium irregulare*	P43	*Camellia alba* 'Plena'	C156	Baiting leaves
ABD.215	MF115340	*Pythium intermedium*	P43	*Camellia alba* 'Plena'	C157	Roots

ABD.217	MF115341	*Pythium dissotocum*	P45	*Viburnum burkwoodii* 'Park Farm hybrid'	C158	Baiting leaves
ABD.218	MF115342	*Pythium dissotocum*	P45	*Viburnum burkwoodii* 'Park Farm hybrid'	N	Baiting leaves
ABD.219	MF115343	*Phytopythium litorale*	P45	*Viburnum burkwoodii* 'Park Farm hybrid'	C161	Roots
ABD.220	MF115344	*Phytophthora cryptogea*	P45	*Viburnum burkwoodii* 'Park Farm hybrid'	N	Roots
ABD.221	MF115345	*Phytophthora taxon PgChlamydo*	P45	*Viburnum burkwoodii* 'Park Farm hybrid'	C162	Roots
ABD.222	MF115346	*Pythium dissotocum*	P45	*Viburnum burkwoodii* 'Park Farm hybrid'	C163	Baiting leaves
ABD.223	MF115347	*Phytophthora taxon PgChlamydo*	P46	*Viburnum burkwoodii* 'Park Farm hybrid'	C164	Roots
ABD.224	MF115348	*Phytophthora cryptogea*	P46	*Viburnum burkwoodii* 'Park Farm hybrid'	C165	Roots
ABD.225	MF115349	*Pythium dissotocum*	P46	*Viburnum burkwoodii* 'Park Farm hybrid'	C166	Roots
ABD.226	MF115350	*Pythium dissotocum*	P46	*Viburnum burkwoodii* 'Park Farm hybrid'	N	Baiting leaves
ABD.228	MF115351	*Phytophthora cryptogea*	P47	*Viburnum burkwoodii* 'Park Farm hybrid'	C169	Roots
ABD.229	MF115352	*Pythium dissotocum*	P47	*Viburnum burkwoodii* 'Park Farm hybrid'	C170	Baiting leaves
ABD.230	MF115353	*Pythium dissotocum*	P47	*Viburnum burkwoodii* 'Park Farm hybrid'	N	Baiting leaves
ABD.231	MF115354	*Pythium dissotocum*	P47	*Viburnum burkwoodii* 'Park Farm hybrid'	N	Baiting leaves
ABD.232	MF115355	*Pythium dissotocum*	P47	*Viburnum burkwoodii* 'Park Farm hybrid'	N	Baiting leaves
ABD.233	MF115356	*Phytophthora taxon PgChlamydo*	P48	*Viburnum burkwoodii* 'Park Farm hybrid'	C174	Roots
ABD.234	MF115357	*Phytophthora cryptogea*	P48	*Viburnum burkwoodii* 'Park Farm hybrid'	C175	Baiting leaves
ABD.235	MF115358	*Pythium dissotocum*	P48	*Viburnum burkwoodii* 'Park Farm hybrid'	C176	Baiting leaves
ABD.236	MF115359	*Pythium dissotocum*	P48	*Viburnum burkwoodii* 'Park Farm hybrid'	C178	Baiting leaves
ABD.237	MF115360	*Phytophthora cryptogea*	P49	*Viburnum burkwoodii* 'Park Farm hybrid'	C180	Roots
ABD.238	MF115361	*Pythium dissotocum*	P49	*Viburnum burkwoodii* 'Park Farm hybrid'	C181	Roots
ABD.240	MF115362	*Phytophthora cryptogea*	P45	*Viburnum burkwoodii* 'Park Farm hybrid'	C159	Baiting leaves
ABD.241	MF115363	*Phytophthora taxon PgChlamydo*	P45	*Viburnum burkwoodii* 'Park Farm hybrid'	C160	Baiting leaves
ABD.242	MF115364	*Pythium dissotocum*	P46	*Viburnum burkwoodii* 'Park Farm hybrid'	N	Roots
ABD.243	MF115365	*Phytophthora taxon PgChlamydo*	P46	*Viburnum burkwoodii* 'Park Farm hybrid'	C167	Baiting leaves
ABD.244	MF115366	*Phytopythium litorale*	P46	*Viburnum burkwoodii* 'Park Farm hybrid'	C168	Baiting leaves
ABD.245	MF115367	*Phytopythium litorale*	P47	*Viburnum burkwoodii* 'Park Farm hybrid'	N	Baiting leaves
ABD.246	MF115368	*Phytophthora cryptogea*	P47	*Viburnum burkwoodii* 'Park Farm hybrid'	C171	Baiting leaves
ABD.247	MF115369	*Phytophthora cryptogea*	P47	*Viburnum burkwoodii* 'Park Farm hybrid'	C172	Substrate
ABD.248	MF115370	*Phytophthora cryptogea*	P47	*Viburnum burkwoodii* 'Park Farm hybrid'	C173	Substrate
ABD.249	MF115371	*Phytophthora taxon PgChlamydo*	P48	*Viburnum burkwoodii* 'Park Farm hybrid'	C177	Baiting leaves
ABD.250	MF115372	*Phytophthora taxon PgChlamydo*	P48	*Viburnum burkwoodii* 'Park Farm hybrid'	C179	Baiting leaves
ABD.251	MF115373	*Phytopythium litorale*	P49	*Viburnum burkwoodii* 'Park Farm hybrid'	C182	Baiting leaves
ABD.252	MF115374	*Phytophthora cryptogea*	P49	*Viburnum burkwoodii* 'Park Farm hybrid'	C183	Substrate
ABD.253	MF115375	*Phytopythium litorale*	P45	*Viburnum burkwoodii* 'Park Farm hybrid'	N	Baiting leaves
ABD.254	MF115376	*Phytophthora cryptogea*	P45	*Viburnum burkwoodii* 'Park Farm hybrid'	C184	Substrate
ABD.255	MF115377	*Phytophthora cryptogea*	P45	*Viburnum burkwoodii* 'Park Farm hybrid'	N	Substrate
ABD.256	MF115378	*Phytophthora taxon PgChlamydo*	P46	*Viburnum burkwoodii* 'Park Farm hybrid'	C185	Substrate
ABD.257	MF115379	*Phytophthora cryptogea*	P46	*Viburnum burkwoodii* 'Park Farm hybrid'	C186	Substrate

ABD.258	MF115380	*Phytophthora cryptogea*	P46	*Viburnum burkwoodii* 'Park Farm hybrid'	N	Substrate
ABD.259	MF115381	*Phytophthora cryptogea*	P46	*Viburnum burkwoodii* 'Park Farm hybrid'	C187	Substrate
ABD.260	MF115382	*Phytopythium litorale*	P47	*Viburnum burkwoodii* 'Park Farm hybrid'	C188	Baiting leaves
ABD.261	MF115383	*Phytopythium litorale*	P48	*Viburnum burkwoodii* 'Park Farm hybrid'	C189	Substrate
ABD.262	MF115384	*Phytophthora cryptogea*	P48	*Viburnum burkwoodii* 'Park Farm hybrid'	C190	Substrate
ABD.263	MF115385	*Phytophthora cryptogea*	P48	*Viburnum burkwoodii* 'Park Farm hybrid'	C191	Substrate
ABD.264	MF115386	*Phytopythium litorale*	P48	*Viburnum burkwoodii* 'Park Farm hybrid'	N	Baiting leaves
ABD.265	MF115387	*Phytophthora cryptogea*	P49	*Viburnum burkwoodii* 'Park Farm hybrid'	C192	Substrate
ABD.266	MF115388	*Phytophthora cryptogea*	P49	*Viburnum burkwoodii* 'Park Farm hybrid'	C193	Substrate
ABD.267	MF115389	*Phytophthora cryptogea*	P49	*Viburnum burkwoodii* 'Park Farm hybrid'	N	Substrate
ABD.268	MF115390	*Pythium sylvaticum*	P50	*Ceanothus thyrsiflorus* 'Repens'	C194	Roots
ABD.269	MF115391	*Pythium sylvaticum*	P50	*Ceanothus thyrsiflorus* 'Repens'	C195	Substrate
ABD.270	MF115392	*Phytophthora cactorum*	P51	*Ceanothus thyrsiflorus* 'Repens'	C196	Roots
ABD.273	MF115393	*Phytophthora cactorum*	P51	*Ceanothus thyrsiflorus* 'Repens'	C199	Substrate
ABD.274	MF115394	*Phytophthora cactorum*	P52	*Ceanothus thyrsiflorus* 'Repens'	C200	Roots
ABD.275	MF115395	*Phytophthora cinnamomi*	P52	*Ceanothus thyrsiflorus* 'Repens'	C201	Substrate
ABD.276	MF115396	*Phytophthora cinnamomi*	P52	*Ceanothus thyrsiflorus* 'Repens'	C202	Substrate
ABD.277	MF115397	*Phytophthora cactorum*	P52	*Ceanothus thyrsiflorus* 'Repens'	C203	Substrate
ABD.278	MF115398	*Phytophthora cactorum*	P52	*Ceanothus thyrsiflorus* 'Repens'	N	Substrate
ABD.283	MF115399	*Phytophthora citrophthora*	P54	*Ceanothus thyrsiflorus* 'Repens'	C204	Roots
ABD.284	MF115400	*Phytophthora cactorum*	P54	*Ceanothus thyrsiflorus* 'Repens'	C205	Substrate
ABD.285	MF115401	*Phytophthora citrophthora*	P54	*Ceanothus thyrsiflorus* 'Repens'	C206	Substrate
ABD.286	MF115402	*Phytophthora cactorum*	P54	*Ceanothus thyrsiflorus* 'Repens'	N	Substrate
ABD.288	MF115403	*Pythium rostratifingens*	P59	*Euonymus fortunei* 'Emerald Gaiety'	C208	Roots
ABD.290	MF115404	*Phytophthora nicotianae*	P50	*Ceanothus thyrsiflorus* 'Repens'	C209	Baiting leaves
ABD.291	MF115405	*Phytophthora nicotianae*	P50	*Ceanothus thyrsiflorus* 'Repens'	C210	Baiting leaves
ABD.292	MF115406	*Phytophthora nicotianae*	P50	*Ceanothus thyrsiflorus* 'Repens'	C211	Baiting leaves
ABD.293	MF115407	*Phytophthora citrophthora*	P51	*Ceanothus thyrsiflorus* 'Repens'	C212	Baiting leaves
ABD.294	MF115408	*Phytophthora cinnamomi*	P51	*Ceanothus thyrsiflorus* 'Repens'	C213	Baiting leaves
ABD.295	MF115409	*Pythium dissotocum*	P51	*Ceanothus thyrsiflorus* 'Repens'	N	Baiting leaves
ABD.296	MF115410	*Phytophthora citrophthora*	P52	*Ceanothus thyrsiflorus* 'Repens'	C214	Baiting leaves
ABD.297	MF115411	*Pythium dissotocum*	P52	*Ceanothus thyrsiflorus* 'Repens'	C215	Baiting leaves
ABD.299	MF115412	*Pythium dissotocum*	P53	*Ceanothus thyrsiflorus* 'Repens'	N	Baiting leaves
ABD.300	MF115413	*Pythium rostratifingens*	P54	*Ceanothus thyrsiflorus* 'Repens'	C216	Baiting leaves
ABD.301	MF115414	*Phytopythium vexans*	P56	*Euonymus fortunei* 'Emerald Gaiety'	C217	Baiting leaves
ABD.302	MF115415	*Pythium dissotocum*	P57	*Euonymus fortunei* 'Emerald Gaiety'	C218	Baiting leaves
ABD.303	MF115416	*Pythium dissotocum*	P50	*Ceanothus thyrsiflorus* 'Repens'	N	Baiting leaves
ABD.304	MF115417	*Phytophthora nicotianae*	P50	*Ceanothus thyrsiflorus* 'Repens'	C219	Baiting leaves
ABD.305	MF115418	*Phytophthora cinnamomi*	P51	*Ceanothus thyrsiflorus* 'Repens'	C220	Baiting leaves

ABD.306	MF115419	*Phytophthora cactorum*	P51	*Ceanothus thyrsiflorus* 'Repens'	C221	Baiting leaves
ABD.307	MF115420	*Phytophthora cactorum*	P51	*Ceanothus thyrsiflorus* 'Repens'	C222	Baiting leaves
ABD.308	MF115421	*Phytophthora cinnamomi*	P52	*Ceanothus thyrsiflorus* 'Repens'	C223	Baiting leaves
ABD.309	MF115422	*Phytophthora cactorum*	P52	*Ceanothus thyrsiflorus* 'Repens'	C224	Baiting leaves
ABD.310	MF115423	*Phytophthora citrophthora*	P53	*Ceanothus thyrsiflorus* 'Repens'	C225	Baiting leaves
ABD.311	MF115424	*Pythium adhaerens*	P54	*Ceanothus thyrsiflorus* 'Repens'	C226	Baiting leaves
ABD.312	MF115425	*Phytophthora nicotianae*	P54	*Ceanothus thyrsiflorus* 'Repens'	C227	Baiting leaves
ABD.313	MF115426	*Phytopythium litorale*	P60	*Ilex meserveae* 'Blue Maid'	N	Baiting leaves
ABD.314	MF115427	*Pythium dissotocum*	P60	*Ilex meserveae* 'Blue Maid'	C229	Baiting leaves
ABD.315	MF115428	*Phytopythium litorale*	P60	*Ilex meserveae* 'Blue Maid'	C228	Substrate
ABD.316	MF115429	*Phytopythium litorale*	P60	*Ilex meserveae* 'Blue Maid'	N	Substrate
ABD.317	MF115430	*Phytophthora cinnamomi*	P61	*Ilex meserveae* 'Blue Maid'	C230	Roots
ABD.318	MF115431	*Phytophthora cinnamomi*	P61	*Ilex meserveae* 'Blue Maid'	N	Baiting leaves
ABD.319	MF115432	*Pythium dissotocum*	P61	*Ilex meserveae* 'Blue Maid'	C231	Baiting leaves
ABD.321	MF115433	*Phytophthora cinnamomi*	P61	*Ilex meserveae* 'Blue Maid'	N	Substrate
ABD.323	MF115434	*Phytophthora cryptogea*	P63	*Ilex meserveae* 'Blue Maid'	C233	Baiting leaves
ABD.325	MF115435	*Phytophthora cryptogea*	P63	*Ilex meserveae* 'Blue Maid'	C234	Substrate
ABD.326	MF115436	*Phytophthora taxon PgChlamydo*	P64	*Ilex aquifolium* 'Argentea'	C235	Roots
ABD.327	MF115437	*Phytophthora taxon PgChlamydo*	P64	*Ilex aquifolium* 'Argentea'	N	Roots
ABD.328	MF115438	*Pythium anandrum*	P64	*Ilex aquifolium* 'Argentea'	C236	Roots
ABD.329	MF115439	*Pythium dissotocum*	P64	*Ilex aquifolium* 'Argentea'	C237	Baiting leaves
ABD.330	MF115440	*Phytopythium litorale*	P64	*Ilex aquifolium* 'Argentea'	C238	Baiting leaves
ABD.332	MF115441	*Pythium anandrum*	P64	*Ilex aquifolium* 'Argentea'	N	Substrate
ABD.333	MF115442	*Phytopythium litorale*	P65	*Ilex aquifolium* 'Argentea'	C239	Roots
ABD.334	MF115443	*Pythium dissotocum*	P65	*Ilex aquifolium* 'Argentea'	C240	Baiting leaves
ABD.336	MF115444	*Pythium dissotocum*	P66	*Ilex aquifolium* 'Argentea'	C241	Baiting leaves
ABD.337	MF115445	*Phytopythium litorale*	P67	*Ilex aquifolium* 'Argentea'	C242	Baiting leaves
ABD.338	MF115446	*Pythium dissotocum*	P67	*Ilex aquifolium* 'Argentea'	C243	Baiting leaves
ABD.339	MF115447	*Phytopythium litorale*	P69	*Ilex altaclerensis* 'Golden King'	C244	Baiting leaves
ABD.340	MF115448	*Phytopythium litorale*	P69	*Ilex altaclerensis* 'Golden King'	C245	Baiting leaves
ABD.341	MF115449	*Phytopythium litorale*	P70	*Ilex altaclerensis* 'Golden King'	C246	Roots
ABD.346	MF115450	*Phytophthora cambivora*	P71	*Ilex altaclerensis* 'Golden King'	C247	Baiting leaves
ABD.347	MF115451	*Phytopythium litorale*	P71	*Ilex altaclerensis* 'Golden King'	C248	Substrate
ABD.348	MF115452	*Phytopythium litorale*	P72	*Pinus mugo*	C249	Roots
ABD.350	MF115453	*Phytopythium litorale*	P72	*Pinus mugo*	C250	Baiting leaves
ABD.352	MF115454	*Phytopythium litorale*	P73	*Pinus mugo*	C251	Roots
ABD.354	MF115455	*Pythium dissotocum*	P73	*Pinus mugo*	C252	Roots
ABD.355	MF115456	*Phytopythium litorale*	P73	*Pinus mugo*	C253	Baiting leaves
ABD.358	MF115457	*Phytopythium litorale*	P74	*Pinus mugo*	C254	Roots

ABD.359	MF115458	*Pythium dissotocum*	P74	*Pinus mugo*	C255	Roots
ABD.360	MF115459	*Phytopythium litorale*	P74	*Pinus mugo*	C256	Baiting leaves
ABD.361	MF115460	*Phytopythium litorale*	P74	*Pinus mugo*	N	Baiting leaves
ABD.365	MF115461	*Phytopythium litorale*	P75	*Pinus mugo*	C257	Roots
ABD.366	MF115462	*Pythium dissotocum*	P75	*Pinus mugo*	C258	Roots
ABD.367	MF115463	*Phytopythium litorale*	P75	*Pinus mugo*	C259	Baiting leaves
ABD.368	MF115464	*Phytopythium litorale*	P76	*Pinus mugo*	C260	Roots
ABD.370	MF115465	*Pythium dissotocum*	P76	*Pinus mugo*	C261	Baiting leaves
ABD.373	MF115466	*Phytopythium litorale*	P77	*Pinus mugo*	C262	Roots
ABD.376	MF115467	*Pythium dissotocum*	P77	*Pinus mugo*	C263	Roots
ABD.377	MF115468	*Pythium dissotocum*	P77	*Pinus mugo*	N	Baiting leaves
ABD.378	MF115469	*Phytopythium litorale*	P77	*Pinus mugo*	N	Baiting leaves
ABD.381	MF115470	*Phytophthora cambivora*	P69	*Ilex altaclerensis* 'Golden King'	C264	Baiting leaves
ABD.383	MF115471	*Phytophthora cambivora*	P71	*Ilex altaclerensis* 'Golden King'	N	Baiting leaves
ABD.386	MF115472	*Phytophthora cambivora*	P73	*Pinus mugo*	C265	Substrate
ABD.389	MF115473	*Phytophthora plurivora*	P60	*Ilex meserveae* 'Blue Maid'	C266	Baiting leaves
ABD.390	MF115474	*Phytophthora plurivora*	P60	*Ilex meserveae* 'Blue Maid'	N	Baiting leaves
ABD.391	MF115475	*Phytophthora cambivora*	P61	*Ilex meserveae* 'Blue Maid'	C267	Baiting leaves
ABD.392	MF115476	*Phytopythium litorale*	P67	*Ilex aquifolium* 'Argentea'	N	Substrate
ABD.399	MF115477	*Phytopythium litorale*	P77	*Pinus mugo*	C268	Baiting leaves
ABD.400	MF115478	*Pythium intermedium*	P99	*Ceanothus thyrsiflorus* 'Southmead'	C269	Roots
ABD.401	MF115479	*Phytophthora cinnamomi*	P99	*Ceanothus thyrsiflorus* 'Southmead'	C270	Baiting leaves
ABD.402	MF115480	*Phytophthora cambivora*	P99	*Ceanothus thyrsiflorus* 'Southmead'	C271	Baiting leaves
ABD.403	MF115481	*Pythium dissotocum*	P99	*Ceanothus thyrsiflorus* 'Southmead'	C272	Baiting leaves
ABD.404	MF115482	*Pythium dissotocum*	P78	*Camellia japonica*	C273	Roots
ABD.405	MF115483	*Pythium dissotocum*	P78	*Camellia japonica*	C274	Baiting leaves
ABD.406	MF115484	*Phytopythium litorale*	P78	*Camellia japonica*	C275	Substrate
ABD.407	MF115485	*Pythium anandrum*	P78	*Camellia japonica*	C276	Substrate
ABD.408	MF115486	*Phytopythium litorale*	P79	*Camellia japonica*	C277	Baiting leaves
ABD.409	MF115487	*Pythium dissotocum*	P79	*Camellia japonica*	C278	Baiting leaves
ABD.411	MF115488	*Phytopythium litorale*	P80	*Camellia japonica*	C279	Baiting leaves
ABD.412	MF115489	*Pythium dissotocum*	P80	*Camellia japonica*	C280	Baiting leaves
ABD.413	MF115490	*Phytopythium litorale*	P81	*Camellia japonica*	C281	Baiting leaves
ABD.414	MF115491	*Pythium dissotocum*	P81	*Camellia japonica*	C282	Baiting leaves
ABD.415	MF115492	*Pythium sylvaticum*	P82	*Buxus sempervirens*	C283	Baiting leaves
ABD.416	MF115493	*Pythium ultimum*	P83	*Buxus sempervirens*	C284	Substrate
ABD.417	MF115494	*Pythium dissotocum*	P84	*Buxus sempervirens*	C285	Baiting leaves
ABD.420	MF115495	*Phytopythium litorale*	P85	*Buxus sempervirens*	C286	Baiting leaves
ABD.421	MF115496	*Phytophthora multivora*	P85	*Buxus sempervirens*	C287	Baiting leaves

ABD.422	MF115497	*Pythium rostratifingens*	P86	*Buxus sempervirens*	C288	Roots
ABD.423	MF115498	*Pythium rostratifingens*	P86	*Buxus sempervirens*	C289	Roots
ABD.424	MF115499	*Pythium dissotocum*	P86	*Buxus sempervirens*	C290	Baiting leaves
ABD.426	MF115500	*Phytopythium litorale*	P88	*Rhododendron obtusum* 'Anouk'	C291	Baiting leaves
ABD.429	MF115501	*Pythium undulatum*	P89	*Rhododendron obtusum* 'Anouk'	C292	Substrate
ABD.430	MF115502	*Phytopythium litorale*	P89	*Rhododendron obtusum* 'Anouk'	C293	Substrate
ABD.431	MF115503	*Phytopythium litorale*	P89	*Rhododendron obtusum* 'Anouk'	N	Substrate
ABD.432	MF115504	*Phytopythium litorale*	P90	*Rhododendron obtusum* 'Anouk'	C294	Substrate
ABD.434	MF115505	*Pythium dissotocum*	P91	*Euonymus fortunei* 'Arlequin'	C295	Baiting leaves
ABD.435	MF115506	*Pythium dissotocum*	P92	*Euonymus fortunei* 'Arlequin'	C296	Baiting leaves
ABD.437	MF115507	*Pythium dissotocum*	P93	*Euonymus fortunei* 'Arlequin'	C297	Roots
ABD.439	MF115508	*Pythium dissotocum*	P94	*Euonymus fortunei* 'Arlequin'	C298	Baiting leaves
ABD.444	MF115509	*Phytopythium litorale*	P96	*Euonymus fortunei* 'Arlequin'	C300	Baiting leaves
ABD.446	MF115510	*Pythium dissotocum*	P96	*Euonymus fortunei* 'Arlequin'	C301	Baiting leaves
ABD.447	MF115511	*Phytophthora multivora*	P96	*Euonymus fortunei* 'Arlequin'	C302	Baiting leaves
ABD.450	MF115512	*Phytopythium litorale*	P97	*Euonymus fortunei* 'Arlequin'	C306	Baiting leaves
ABD.452	MF115513	*Phytopythium citrinum*	P97	*Euonymus fortunei* 'Arlequin'	C307	Baiting leaves
ABD.455	MF115514	*Pythium dissotocum*	P98	*Euonymus fortunei* 'Arlequin'	C308	Baiting leaves
ABD.456	MF115515	*Phytophthora multivora*	P98	*Euonymus fortunei* 'Arlequin'	C309	Baiting leaves
ABD.457	MF115516	*Phytophthora citrophthora*	P98	*Euonymus fortunei* 'Arlequin'	C310	Baiting leaves
ABD.458	MF115517	*Phytophthora plurivora*	P98	*Euonymus fortunei* 'Arlequin'	C311	Baiting leaves
ABD.459	MF115518	*Phytophthora plurivora*	P98	*Euonymus fortunei* 'Arlequin'	N	Baiting leaves
ABD.461	MF115519	*Pythium undulatum*	P98	*Euonymus fortunei* 'Arlequin'	C312	Baiting leaves
ABD.463	MF115520	*Phytophthora citrophthora*	P98	*Euonymus fortunei* 'Arlequin'	N	Baiting leaves
ABD.464	MF115521	*Phytophthora multivora*	P98	*Euonymus fortunei* 'Arlequin'	N	Baiting leaves
ABD.465	MF115522	*Phytophthora citrophthora*	P96	*Euonymus fortunei* 'Arlequin'	C303	Baiting leaves
ABD.466	MF115523	*Phytopythium citrinum*	P96	*Euonymus fortunei* 'Arlequin'	C304	Baiting leaves
ABD.467	MF115524	*Phytophthora multivora*	P95	*Euonymus fortunei* 'Arlequin'	C305	Baiting leaves

Table S2. eDNA quantification with ITS, trnM-trnP-trnM and atp9-nad9 probes (in ng g^{-1} of plant compost, roots or crushed filters), and list of oomycete species isolated on each plant using classical methods. *: Asymptomatic plant. blank: no amplification. N: negative sample.

**Plant**	**ITS**			**trnM**			atp9			Oomycete species isolated by classical techniques and baitings	
	**Filters**	**Roots**	**Compost**	**Filters**	**Roots**	**Compost**	**Filters**	**Roots**	**Compost**	***Phytophthora* spp.**	***Pythium & Phytopythium* spp.**
P1	$2.26\ 10^{-1}$	5.82	$3.65\ 10^{-1}$		$7.14\ 10^{-2}$	$3.66\ 10^{-2}$	$1.10\ 10^{-1}$	2.28	$5.48\ 10^{-3}$	*P. cryptogea, P. ramorum*	*P. dissotocum, P. litorale, P. lutarium*
P2	$3.59\ 10^{-2}$	$9.90\ 10^{-2}$	18.05					$3.61\ 10^{-4}$		-	*P. citrinum, P. chamaehyphon, P. dissotocum, P. irregulare*
P3	$4.85\ 10^{-1}$	$2.02\ 10^{-2}$	$6.60\ 10^{-1}$	$1.65\ 10^{-1}$			$1.02\ 10^{-2}$	$5.08\ 10^{-5}$		-	*P. undulatum*
P4	$2.27\ 10^{-1}$	$3.15\ 10^{-2}$	$2.18\ 10^{-1}$	$4.70\ 10^{-2}$			$1.80\ 10^{-2}$		$5.49\ 10^{-3}$	*P. plurivora*	*P. debaryanum/violae*
P5	1.21	$1.89\ 10^{-2}$	$1.63\ 10^{-1}$	$9.91\ 10^{-3}$			$5.55\ 10^{-2}$			-	-
P6	$8.92\ 10^{-1}$	$2.15\ 10^{-2}$	$3.40\ 10^{-1}$	$5.14\ 10^{-2}$		$8.72\ 10^{-3}$	$2.35\ 10^{-2}$	N		-	*P. torulosum/catenulatum, P. undulatum*
P7	$2.00\ 10^{-1}$	$1.24\ 10^{-2}$	1.53	$3.51\ 10^{-2}$			$2.84\ 10^{-2}$	$6.30\ 10^{-4}$	$3.21\ 10^{-4}$	-	*P. intermedium/sylvaticum*
*P8	4.17	$1.74\ 10^{-2}$								-	*P. chamaehyphon, P. dissotocum*
*P9	1.07	$3.85\ 10^{-3}$	14.19							-	*P. chamaehyphon, P. dissotocum, P. litorale*
*P10		$1.02\ 10^{-2}$								-	*P. chamaehyphon, P. diclinum, P. dissotocum, P. vexans*
*P11	$3.57\ 10^{-2}$	$4.01\ 10^{-2}$								-	*P. diclinum, P. dissotocum, P. litorale*
*P12	$2.35\ 10^{-2}$	$1.12\ 10^{-2}$								-	*P. chamaehyphon, P. dissotocum*
*P13	$5.22\ 10^{-2}$	$1.25\ 10^{-2}$	1.47							-	*P. chamaehyphon, P. dissotocum*
*P14	1.49	1.83	29.62	$2.86\ 10^{-1}$	$1.79\ 10^{-2}$	9.91	$4.01\ 10^{-1}$	$2.01\ 10^{-1}$	6.59	*P. cinnamomi*	*P. undulatum*
*P15	$2.29\ 10^{-1}$	$1.31\ 10^{-1}$	47.81	$7.19\ 10^{-2}$	$2.14\ 10^{-4}$	4.43	$1.20\ 10^{-1}$	$5.68\ 10^{-3}$	2.66	*P. cinnamomi*	*P. undulatum*
*P16	$1.03\ 10^{-1}$	$7.41\ 10^{-2}$	14.88			$9.04\ 10^{-3}$			$9.19\ 10^{-3}$	-	*P. helicoides, P. macrosporum*
*P17	$1.46\ 10^{-1}$	$1.18\ 10^{-1}$	28.62			$1.09\ 10^{-2}$			$4.22\ 10^{-4}$	-	*P. macrosporum, P. undulatum*
*P18	$2.24\ 10^{-1}$	$2.14\ 10^{-2}$	22.73			$4.24\ 10^{-2}$			$3.88\ 10^{-2}$	-	*P. undulatum*
*P19	$2.86\ 10^{-1}$	$1.34\ 10^{-2}$	16.35							-	*P. undulatum*
P20	$3.71\ 10^{-1}$	$2.44\ 10^{-1}$	8.95		$7.24\ 10^{-4}$	$2.03\ 10^{-2}$		$7.00\ 10^{-2}$	$5.00\ 10^{-2}$	*P. cambivora*	*P. dissotocum*
P21	$1.50\ 10^{-1}$	1.51	42.05		$1.90\ 10^{-3}$	$1.37\ 10^{-1}$	$4.35\ 10^{-2}$	$5.48\ 10^{-1}$	$3.05\ 10^{-1}$	*P. cambivora, P. plurivora*	*P. dissotocum*
P22	$9.17\ 10^{-2}$	1.48	191.34		$9.25\ 10^{-2}$	$6.06\ 10^{-2}$		1.03	$1.71\ 10^{-1}$	*P. cambivora*	*P. dissimile/pyrilobum, P. dissotocum*
P23	$2.94\ 10^{-1}$	1.28	34.31		$2.79\ 10^{-2}$	$5.64\ 10^{-2}$		$5.51\ 10^{-1}$	$1.63\ 10^{-1}$	*P. cambivora*	*P. dissotocum, P. intermedium*
P24	$3.08\ 10^{-1}$	$4.93\ 10^{-1}$	16.70		$2.60\ 10^{-3}$	$1.16\ 10^{-2}$		$2.60\ 10^{-1}$	$8.64\ 10^{-2}$	*P. cambivora*	*P. dissotocum*
P25	$1.56\ 10^{-1}$	$1.20\ 10^{-2}$	13.61							-	-
P26	$2.78\ 10^{-1}$	$1.16\ 10^{-2}$	14.56			$1.87\ 10^{-2}$			$1.42\ 10^{-2}$	-	*P. dissotocum*
P27	$1.95\ 10^{-1}$	$2.37\ 10^{-2}$	14.53							-	*P. dissotocum, P. intermedium*
P28	$6.42\ 10^{-2}$	$5.10\ 10^{-3}$	12.08				$3.01\ 10^{-3}$		$1.08\ 10^{-2}$	-	*P. deliense, P. dissotocum*
P29	$1.10\ 10^{-1}$		12.20							-	*P. helicoides, P. kashmirense, P. sterilum*
P30	$8.73\ 10^{-2}$	$2.65\ 10^{-2}$	14.23			$6.70\ 10^{-2}$	$2.92\ 10^{-4}$		$2.62\ 10^{-3}$	*P. chlamydospora,*	*P. dissotocum, P. litorale*

										P. cryptogea	
P31	$1.07\ 10^{-1}$	$2.46\ 10^{-2}$	9.35			$2.56\ 10^{-1}$			$9.27\ 10^{-3}$	*P. cryptogea*	*P. dissotocum, P. litorale*
P32	$7.99\ 10^{-2}$	$9.23\ 10^{-3}$	12.71			$3.86\ 10^{-2}$		N		*P. cryptogea*	*P. dissotocum*
P33	$1.45\ 10^{-1}$	$1.61\ 10^{-1}$	12.89	$3.57\ 10^{-4}$		$1.56\ 10^{-1}$			$5.78\ 10^{-2}$	*P. chlamydospora, P. cryptogea*	*P. anandrum*
P34	$1.11\ 10^{-1}$	$9.94\ 10^{-2}$	8.17			$3.61\ 10^{-2}$			$6.46\ 10^{-3}$	*P. chlamydospora, P. cactorum, P. cryptogea*	*P. dissotocum, P. litorale*
P35	$1.28\ 10^{-1}$	$1.20\ 10^{-2}$	11.50			$3.86\ 10^{-2}$		$4.13\ 10^{-4}$	$1.25\ 10^{-1}$	*P. cactorum, P. citrophthora*	*P. vexans*
P36	$1.93\ 10^{-1}$	$2.15\ 10^{-3}$	1.03			$4.35\ 10^{-2}$	$1.21\ 10^{-1}$		$3.15\ 10^{-1}$	*P. citrophthora*	*P. dissotocum*
P37	$4.43\ 10^{-1}$	$8.17\ 10^{-3}$	$3.99\ 10^{-1}$			$2.11\ 10^{-1}$			$1.57\ 10^{-2}$	*P. cactorum*	*P. chamaehyphon, P. helicoides*
P38	$2.37\ 10^{-1}$	$3.61\ 10^{-3}$	1.91	$4.61\ 10^{-2}$		3.21	$9.42\ 10^{-2}$	$4.94\ 10^{-4}$	2.75	*P. citrophthora, P. cryptogea*	*P. dissotocum*
P39	$1.61\ 10^{-1}$	$2.47\ 10^{-3}$	$9.41\ 10^{-1}$	$8.93\ 10^{-2}$	$4.98\ 10^{-5}$	$5.43\ 10^{-1}$	$2.00\ 10^{-1}$	$1.81\ 10^{-3}$	$2.69\ 10^{-1}$	*P. citrophthora, P. cryptogea*	*P. chamaehyphon, P. dissotocum*
*P40	$2.47\ 10^{-2}$	$6.91\ 10^{-3}$	$4.95\ 10^{-2}$							-	-
*P41	$1.43\ 10^{-2}$	$3.86\ 10^{-2}$	$3.18\ 10^{-2}$							-	-
*P42	$2.80\ 10^{-2}$	$5.05\ 10^{-3}$	$7.55\ 10^{-2}$							-	-
*P43		$3.91\ 10^{-2}$	$3.62\ 10^{-2}$							-	*P. heterothallicum, P. intermedium, P. irregulare*
*P44	$2.49\ 10^{-2}$	$6.00\ 10^{-2}$	$1.13\ 10^{-1}$							-	-
P45		$2.47\ 10^{-2}$	$3.21\ 10^{-2}$							*P. chlamydospora, P. cryptogea*	*P. dissotocum, P. litorale*
P46	$5.82\ 10^{-2}$	$1.19\ 10^{-1}$	$2.49\ 10^{-2}$	$1.54\ 10^{-3}$			$1.72\ 10^{-2}$			*P. chlamydospora, P. cryptogea*	*P. dissotocum, P. litorale*
P47	$1.15\ 10^{-1}$	$1.64\ 10^{-1}$	$4.10\ 10^{-2}$				$2.16\ 10^{-3}$			*P. cryptogea*	*P. dissotocum, P. litorale*
P48		$7.11\ 10^{-2}$	$1.67\ 10^{-1}$					$3.09\ 10^{-3}$	$3.24\ 10^{-2}$	*P. chlamydospora, P. cryptogea*	*P. dissotocum, P. litorale*
P49	$1.34\ 10^{-2}$	$2.38\ 10^{-2}$	$3.26\ 10^{-2}$			$2.45\ 10^{-2}$				*P. cryptogea*	*P. dissotocum, P. litorale*
*P50	$1.25\ 10^{-1}$	$3.28\ 10^{-1}$	2.20			$1.50\ 10^{-1}$		$6.98\ 10^{-3}$		*P. nicotianae*	*P. lutarium, P. sylvaticum*
*P51	$2.30\ 10^{-1}$	$3.20\ 10^{-1}$	1.12	$1.38\ 10^{-2}$		$1.52\ 10^{-1}$	$7.20\ 10^{-2}$	$1.88\ 10^{-2}$	$4.06\ 10^{-1}$	*P. cactorum, P. cinnamomi, P. citrophthora*	*P. dissotocum, P. sylvaticum/terrestris*
*P52	$2.72\ 10^{-1}$	$7.38\ 10^{-1}$	$4.33\ 10^{-1}$	$2.68\ 10^{-2}$	$1.70\ 10^{-4}$	$7.35\ 10^{-2}$	$1.35\ 10^{-1}$	$2.17\ 10^{-1}$	$1.78\ 10^{-1}$	*P. cactorum, P. cinnamomi, P. citrophthora*	*P. dissotocum*
*P53	$2.00\ 10^{-2}$	$5.13\ 10^{-2}$	$1.83\ 10^{-1}$			$2.48\ 10^{-2}$		$1.27\ 10^{-2}$	$8.78\ 10^{-2}$	*P. citrophthora*	*P. dissotocum*
*P54	$3.91\ 10^{-2}$	$1.27\ 10^{-1}$	$1.98\ 10^{-1}$	$1.57\ 10^{-3}$	N	$2.78\ 10^{-2}$		$2.75\ 10^{-2}$	$5.31\ 10^{-2}$	*P. cactorum, P. citrophthora, P. nicotianae*	*P. adhaerens/chondricola*

P55	4.27 10^{-2}	6.76 10^{-4}	5.67 10^{-2}							-	-
P56	4.90 10^{-1}	7.91 10^{-4}	2.50 10^{-2}						2.79 10^{-2}	-	*P. vexans*
P57	2.73 10^{-2}	6.90 10^{-3}	5.80 10^{-2}							-	*P. dissotocum*
P58	4.80 10^{-2}	6.88 10^{-4}								-	-
P59	8.69 10^{-2}	1.96 10^{-3}	7.03 10^{-2}							-	*P. rostratifingens*
P60	1.52 10^{-1}		2.80				1.63 10^{-2}		1.27 10^{-2}	*P. plurivora*	*P. dissotocum, P. litorale*
P61	2.85 10^{-1}	10.94	15.45	1.60 10^{-3}		3.73 10^{-1}	5.10 10^{-2}		2.53	*P. cambivora, P. cinnamomi, Phytophthora sp.*	*P. citrinum, P. dissotocum*
P62	3.44 10^{-1}	1.92 10^{-1}	6.51 10^{-1}						1.12 10^{-1}	*Phytophthora* sp.	-
P63	1.65 10^{-1}	7.14 10^{-2}	2.34 10^{-1}						1.76 10^{-2}	*P. cryptogea, Phytophthora* spp.	-
*P64	5.29 10^{-1}	3.25 10^{-2}	2.28	6.88 10^{-3}					2.58 10^{-2}	*P. chlamydospora, Phytophthora* spp.	*P. anandrum, P. dissotocum, P. litorale*
P65	1.97 10^{-1}	5.14 10^{-2}	2.60 10^{-1}						8.27 10^{-3}	-	*P. dissotocum, P. litorale*
P66	4.48 10^{-1}	3.83 10^{-3}	4.32 10^{-1}						8.82 10^{-4}	*Phytophthora* spp.	*P. dissotocum*
*P67	1.81 10^{-1}	2.69 10^{-3}	1.21							-	*P. dissotocum, P. litorale*
*P68	7.48 10^{-2}	1.67 10^{-3}	3.04 10^{-1}							-	-
P69	1.17 10^{-1}	8.78 10^{-3}	5.87 10^{-1}					9.52 10^{-4}	6.71 10^{-3}	*P. cambivora*	*P. litorale/sterilum*
P70	2.40 10^{-1}	3.37 10^{-3}	3.87 10^{-1}					1.21 10^{-4}		*Phytophthora* spp.	*P. litorale/sterilum*
P71	7.71 10^{-2}	1.14 10^{-2}	1.58 10^{-1}	2.01 10^{-4}			2.41 10^{-2}	N	1.43 10^{-3}	*P. cambivora, Phytophthora* spp.	*P. litorale/sterilum*
*P72	1.08	1.90	8.22	7.36 10^{-3}	8.94 10^{-3}	1.27 10^{-1}	1.56 10^{-1}	2.53 10^{-1}	1.01	*Phytophthora* spp.	*P. litorale/sterilum*
*P73	2.44 10^{-1}	1.12	5.04	4.46 10^{-3}	7.77 10^{-3}	3.19 10^{-1}	1.49 10^{-1}	1.73 10^{-1}	2.85	*P. cambivora*	*P. dissotocum, P. litorale/sterilum*
*P74	4.52 10^{-1}	1.69	8.99		5.83 10^{-3}	8.70 10^{-2}	1.51 10^{-1}	1.98 10^{-1}	1.65	*Phytophthora* spp.	*P. dissotocum, P. litorale/sterilum*
*P75	5.93 10^{-1}	1.96	8.35	4.00 10^{-2}	9.94 10^{-1}	8.09 10^{-1}	1.83 10^{-1}	4.12 10^{-1}	1.65	-	*P. dissotocum, P. litorale/sterilum*
*P76	6.28 10^{-1}	3.29 10^{-1}	4.58	3.36 10^{-2}	2.74 10^{-2}	5.70 10^{-1}	1.24 10^{-1}	7.06 10^{-2}	8.59 10^{-1}	-	*P. dissotocum, P. litorale/sterilum*
*P77	5.66 10^{-1}	9.90 10^{-1}	8.24	2.04 10^{-1}	8.32 10^{-2}	3.42	4.11 10^{-1}	2.09 10^{-1}	5.74	-	*P. dissotocum, P. litorale*
P78	1.77 10^{-2}	2.74 10^{-1}	3.70 10^{-1}				2.78 10^{-2}	2.71 10^{-2}	1.17 10^{-1}	-	*P. anandrum, P. dissotocum, P. litorale*
P79	1.48 10^{-2}	4.19 10^{-1}	2.27	7.55 10^{-4}	3.98 10^{-3}	4.36 10^{-2}	1.56 10^{-2}	5.15 10^{-2}	8.11 10^{-2}	-	*P. dissotocum, P. litorale*
P80		2.15 10^{-1}	2.22 10^{-1}		1.01 10^{-3}		4.30 10^{-3}	5.55 10^{-2}	3.64 10^{-2}	-	*P. dissotocum, P. litorale*
P81	1.14 10^{-2}	3.12 10^{-1}	5.16 10^{-1}		N	1.71 10^{-2}		1.94 10^{-2}	7.39 10^{-2}	-	*P. dissotocum, P. litorale*
P82										-	*P. sylvaticum*
P83	1.84 10^{-2}	2.27 10^{-1}	7.87 10^{-2}							-	*P. ultimum var. ultimum*
P84	4.31 10^{-2}	3.29 10^{-3}								-	*P. dissotocum*
P85	3.57 10^{-2}	4.10 10^{-1}	3.22 10^{-1}							*P. multivora*	*P. litorale*
P86		1.44 10^{-1}	1.97 10^{-1}							-	*P. dissotocum, P. rostratifingens*
P87	3.35 10^{-2}	2.55 10^{-3}	1.57 10^{-1}							-	-
P88	2.52 10^{-2}	1.66 10^{-2}	2.16 10^{-1}							-	*P. litorale*

P89	$4.47\ 10^{-3}$	$8.21\ 10^{-2}$	$3.96\ 10^{-2}$		$1.96\ 10^{-2}$			$2.48\ 10^{-2}$		-	*P. litorale, P. ultimum*
P90	$5.23\ 10^{-3}$	$2.01\ 10^{-3}$								-	*P. litorale*
*P91	$3.43\ 10^{-2}$	$6.52\ 10^{-1}$		$2.57\ 10^{-3}$	$5.58\ 10^{-2}$		$4.58\ 10^{-2}$	$3.86\ 10^{-1}$	$6.19\ 10^{-2}$	-	*P. dissotocum*
*P92		$5.34\ 10^{-1}$	$1.86\ 10^{-1}$	$7.89\ 10^{-4}$	$9.72\ 10^{-3}$			$2.16\ 10^{-1}$		-	*P. dissotocum*
*P93	$3.87\ 10^{-2}$	$1.75\ 10^{-1}$	$1.79\ 10^{-1}$		$5.27\ 10^{-3}$		$1.25\ 10^{-2}$	$9.22\ 10^{-2}$	$3.10\ 10^{-2}$	-	*P. dissotocum*
*P94	$8.56\ 10^{-2}$	$4.92\ 10^{-1}$	$1.13\ 10^{-1}$		$9.08\ 10^{-3}$		$6.61\ 10^{-2}$	$1.88\ 10^{-1}$	$4.15\ 10^{-2}$	-	*P. dissotocum*
P95		$1.65\ 10^{-1}$	$2.53\ 10^{-1}$	$5.77\ 10^{-4}$				$4.04\ 10^{-2}$	$2.48\ 10^{-2}$	*P. multivora*	-
P96		$4.96\ 10^{-1}$	$2.02\ 10^{-1}$			$1.03\ 10^{-3}$		$7.82\ 10^{-2}$	$3.96\ 10^{-2}$	*P. citrophthora, P. multivora*	*P. citrinum, P. dissotocum, P. litorale*
P97	$8.13\ 10^{-2}$	$5.08\ 10^{-1}$	$6.83\ 10^{-1}$		$6.10\ 10^{-3}$		$4.13\ 10^{-2}$	$1.51\ 10^{-1}$	$5.83\ 10^{-2}$	-	*P. citrinum, P, litorale*
P98		$3.13\ 10^{-1}$	$3.99\ 10^{-1}$				$1.69\ 10^{-2}$	$3.96\ 10^{-2}$	$3.38\ 10^{-2}$	*P. citrophthora, P. multivora, P. plurivora*	*P. dissotocum, P. undulatum, P. vexans, P. ultimum*
P99	1.63	$5.32\ 10^{-1}$	1.61	2212.20	$3.56\ 10^{-2}$	$6.15\ 10^{-1}$	6.03	$1.06\ 10^{-1}$	$5.20\ 10^{-1}$	*P. cambivora, P. cinnamomi*	*P. dissotocum, P. intermedium*
